# scDual-Seq of *Toxoplasma gondii*-infected mouse BMDCs reveals heterogeneity and differential infection dynamics

**DOI:** 10.3389/fimmu.2023.1224591

**Published:** 2023-07-27

**Authors:** Franziska Hildebrandt, Mubasher Mohammed, Alexis Dziedziech, Amol K. Bhandage, Anna-Maria Divne, Fredrik Barrenäs, Antonio Barragan, Johan Henriksson, Johan Ankarklev

**Affiliations:** ^1^ Department of Molecular Biosciences, The Wenner Gren Institute, Stockholm University, Stockholm, Sweden; ^2^ Department of Global Health, Institut Pasteur, Paris, France; ^3^ Microbial Single Cell Genomics Facility, SciLifeLab, Biomedical Center (BMC) Uppsala University, Uppsala, Sweden; ^4^ Laboratory of Molecular Infection Medicine Sweden (MIMS), Umeå Center for Microbial Research, Department of Molecular Biology, Umeå University, Umeå, Sweden

**Keywords:** *Toxoplasma gondii*, bone marrow-derived dendritic cells, BMDCs, host-pathogen interactions, immune modulation, scDual-Seq, Dual single-cell RNA-seq

## Abstract

Dendritic cells and macrophages are integral parts of the innate immune system and gatekeepers against infection. The protozoan pathogen, *Toxoplasma gondii*, is known to hijack host immune cells and modulate their immune response, making it a compelling model to study host-pathogen interactions. Here we utilize single cell Dual RNA-seq to parse out heterogeneous transcription of mouse bone marrow-derived dendritic cells (BMDCs) infected with two distinct genotypes of *T. gondii* parasites, over multiple time points post infection. We show that the BMDCs elicit differential responses towards *T. gondii* infection and that the two parasite lineages distinctly manipulate subpopulations of infected BMDCs. Co-expression networks define host and parasite genes, with implications for modulation of host immunity. Integrative analysis validates previously established immune pathways and additionally, suggests novel candidate genes involved in host-pathogen interactions. Altogether, this study provides a comprehensive resource for characterizing host-pathogen interplay at high-resolution.

## Introduction

Infection dynamics are determined by the interactions between an infectious agent, such as viruses, bacteria and parasites and their respective host. The host cells are required to recognize and respond to the intruder through activation of the immune system, while the invading microbes have evolved multiple strategies to ensure their survival, including evasion of the host’s immune response. Many obligate intracellular microorganisms are found to alter the cellular expression programs of infected and surrounding cells to actively evade immunity, scavenge nutrients and establish chronic infection or dormancy states within the host. Conversely, host cells have developed numerous protective mechanisms to prevent the establishment of different pathogens (1). Host cell responses can be unique among different host cell types but also towards different types or sub-types of pathogens.

The mammalian immune system heavily relies on innate immune sensors important in microbial detection (2). These sensors are commonly referred to as pattern recognition receptors (PRRs) and include a variety of different receptor types with the ability to recognize a wide-range of pathogen associated molecular Patterns (PAMPs). The most prominent PRR family comprises Toll-like receptors (TLRs), which can be present in the extracellular- or endosomal membrane of innate immune cells (3, 4). Two cell types of importance are the dendritic cells (DCs) and macrophages, which both present antigens to the adaptive immune cells upon recognition of a pathogen. DCs and macrophages are among the first responders to an infection as part of the mononuclear phagocyte system (5).Both cell types share the ability to produce cytokines upon stimulation leading to the initiation of an immune response. DCs have superior antigen presenting capacities and migrate to secondary lymphoid organs where they present antigen to naive T cells, resulting in their activation. macrophages on the other hand are considered general effector cells, presenting antigen at the site of infection and are primarily known for their role in phagocytosis and production of anti-inflammatory cytokines (6–8).

Distinct subtypes of bone marrow-derived DCs (BMDCs) have been described, including cells which display expression profiles characteristic of macrophages. In fact, Helft and colleagues used a sophisticated FACS approach for enrichment of subpopulation followed by gene expression analysis, to define that CD11c+ BMDCs comprise a heterogeneous population of macrophages and DCs (9). These subtypes have also been shown to display specific functions in response to particular pathogens or pathogen derived factors (5, 9, 10). For example, LPS was found to elicit a bimodal transcriptional response in two subpopulations of BMDCs (9, 10).

Certain intracellular parasites have become highly successful in transmission and in establishing chronic infection, as evidenced by their global prevalence. However, the understanding of the dynamics of host-pathogen interactions among these parasites remains fragmented. The extent of heterogeneity within BMDCs and other immune cell populations in their steady-state and in response to pathogens who are known immune modulators, such as *T. gondii*, has yet to be explored extensively at cellular resolution.

*Toxoplasma gondii* belongs to the Apicomplexan phylum including more than 4500 species of obligate intracellular, parasitic protozoa (11). Uniquely, *T. gondii* demonstrates the highest prevalence of infection among all known parasitic diseases of humankind, where one third of the human population is estimated to be infected (12). Infection occurs by ingestion of undercooked meat containing parasite cysts or *via* consumption of contaminated food or water containing oocysts originating from feline feces (13, 14). The majority of individuals infected with *Toxoplasma* are asymptomatic. However, vulnerable individuals including the immunocompromised, pregnant women and children born from an infected mother, can experience severe or even lethal disease in the form of disseminated or latent infections or cerebral toxoplasmosis (14).

An active infection is typically characterized by rapidly replicating tachyzoites, able to invade any nucleated host cell. The interaction between parasite-secreted proteins from organelles such as rhoptries, micronemes and dense granules within the apical complex and host adhesion receptors, for example host β-tubulin, galactose-containing carbohydrate ligands or sialic acids, aid in the invasion process (15, 16). Extensive and continuous replication of intracellular tachyzoites eventually leads to the disruption of the host cell. Thereafter parasites invade surrounding cells or disseminate to other areas of the body, even crossing barriers into the placenta or the brain (17).

DCs and macrophages, which are enriched in the intestinal lamina propria and the Peyer’s patches are the first responding immune cells towards incoming *Toxoplasma* parasites (18). The parasite can modulate the migratory behavior (19–22) or the immune response of infected macrophages and DCs for example to migrate to distant organs, including the brain. There, the parasite can then establish chronic infection through the formation of bradyzoites (23).

Across Europe and North America, the predominant infecting clonal lineages, i.e. *T. gondii* subpopulations which are related by descent, are commonly separated into type I, type II and type III parasites (24). They are not considered true species as sexual recombination, albeit infrequently, may occur between them (25, 26). These clonal lineages show differential correlations with disease progression and outcome in both mice and humans (25, 27). Type I parasites, as opposed to type II or type III parasites, cause lethal virulence in mice (27). In contrast, type II parasites disseminate more effectively due to increased hypermotility and longer migratory distances in infected leukocytes, possibly giving them an advantage in the development of chronic infection (28). Further, clonal lineages display differences in macrophage activation, with type II parasites activating classical immune activation, while type I and type III parasites induce alternative macrophage activation (29).

Previous characterization of transcriptional changes upon infection with *T. gondii* was integral to gain insight in the host-response. However, current publicly available data is mostly limited to population-wide bulk RNA sequencing (30, 31) of the host or in tandem with the parasite (32) as well as single cell RNA sequencing data of the host alone (33). Single cell dual sequencing (sc-DualSeq) enables dissecting and deciphering molecular population heterogeneity during highly dynamic interactions of an active infection (34, 35). The applicability of sc-DualSeq and the relevance of heterogeneity in infection dynamics was recently demonstrated in *T. gondii* infected monocytes (36) and human foreskin fibroblasts (37). However, an exhaustive image of host-pathogen interactions between different *T. gondii* clonal lineages and murine leukocytes on the transcriptional level and in single cells over other relevant time points has yet to be described.

In this study we aim to parse out the heterogeneity of complex host-parasite interplay by implementing scDual-seq to explore the interactions between murine BMDCs infected with type I and II *T. gondii* parasites over time, to identify inter-species co-expression networks.

We further aim to uncover underlying factors of heterogeneity in the host cell population beyond inherent stochasticity of gene expression, such as hidden cell subtypes and the effects of parasite secretion of effector molecules on the host cell. Additionally, we aim to create a computational resource to compare transcriptional responses in host-pathogen complexes, with the ambition of enabling the broader infection and immunology community to explore similarities between gene expression profiles in response to *T. gondii* infection and other host-pathogen interactions. This may include but is not limited to other protozoan pathogens, such as *Plasmodium spp*, *Cryptosporidum spp*, and *Leishmania* spp.

## Results

### *T. gondii* infects two sub-populations of bone marrow-derived dendritic cells

**Figure 1:**
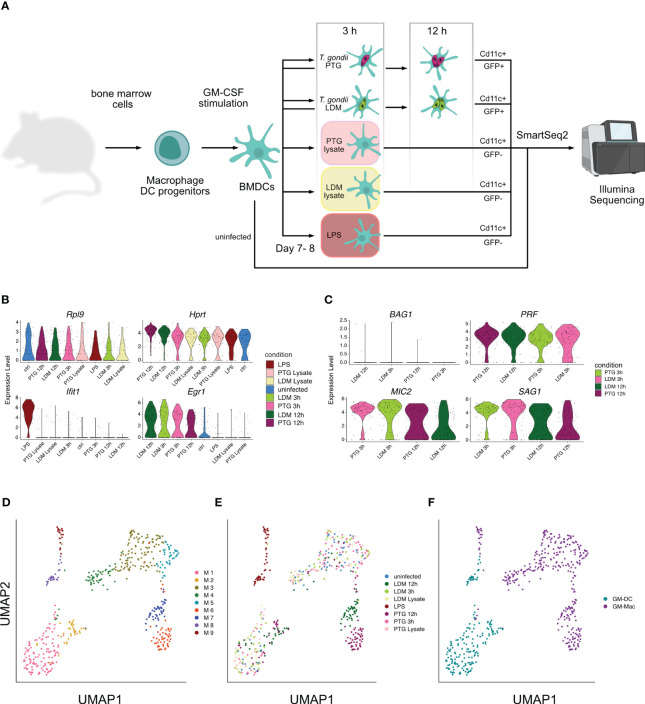
Transcriptional analysis of Toxoplasma BMDC infection at the single cell level. **(A)** Schematic overview of study design and biological significance. Primary BMDCs were collected, activated and infected with *T. gondii* LDM (LDM) and PTG (PTG) for 3 and 12 hours. Heat-inactivated lysates and LPS served as controls. Cells were then sorted based on GFP+ fluorescence from the parasite and presence of the CD11C-PE-Cy7 surface marker and subjected to scRNA-seq and subsequent analysis. In part created with https://www.biorender.com/. **(B)** Distribution of expression levels across infection conditions of selected mouse marker genes ordered by expression levels from highest to lowest. **(C)** Distribution of selected *T. gondii* markers across original conditions ordered by expression levels from highest to lowest. **(D)** UMAP projection of single cells colored by identified clusters from graph-based clustering. **(E)** UMAP projection of single cells colored by infection condition. **(F)** UMAP projection of single cells colored by infected host cell type.

We recovered transcripts of host and pathogen signatures from individual BMDCs infected with two different strains of *T. gondii* parasites: *T. gondii* PTG (ME49-PTG, clonal type II) and *T. gondii* LDM (RH-LDM, clonal type I) at 3 and 12 hours post infection (hpi). Between these time points, the parasites undergo at least one cycle of endodyogeny, a specialized replication mechanism of *T. gondii* tachyzoites (38), leading to two or four parasites within each host cell at 12 hpi. Given that parasite lysate, consists of inactive parasites, the ability of active invasion and intracellular host cell modulation over time is lost, we only collected data for all controls at 3 hpi (**Figure 1A**).

The majority of reads originated from host transcripts across all cells and *T. gondii* PTG transcripts decrease between 3 and 12 hpi, as opposed to an increase of transcripts for LDM parasites. This indicates time-dependent differences in general transcriptional activity between strains (**Supplementary Figure 1**).

To validate our approach, we inspected the distribution of established host- and parasite marker genes across conditions. Housekeeping genes *Rpl9* and *Hprt* (39, 40) show equal expression levels across all conditions and cells. *Ifit1*, a marker for immune response to LPS infection in macrophages (41), is upregulated in LPS-stimulated cells but not in *T. gondii* infected cells. Conversely, only cells infected with *T. gondii* sustain previously reported high levels of *Egr1* expression (42), independent of their clonal lineage (**Figure 1B**).

The expression of the *T. gondii* marker gene, *BAG1*, exclusively expressed in bradyzoite stages (43), showed no upregulation in our data. *PRF*, essential for gliding motility, invasion and exit from host cells (44), shows increased expression in the late stages of *T. gondii* tachyzoites. *MIC2* and *SAG1*, two other *T. gondii* genes expressing proteins essential for invasion, virulence and host cell binding (45, 46), showed expression across all infection conditions, peaking at early time points (**Figure 1C**). The observed expression of these established host and T. gondii marker genes in all cells of their respective condition justifies the number of cells used here, to investigate host-pathogen interactions.

For each infected cell, we retrieved expression data from the host and the parasite. We then isolated expression values of the host and the parasite to analyze them individually. Unsupervised clustering of the host data resulted in 9 clusters (M1 - M9) across all conditions (**Figure 1D**). Comparing the cluster annotations of cells to the experimental conditions in a UMAP embedding, showed that cluster M8 and M9 were comprised of only LPS activated cells, while cluster M2 and M6-M7 were comprised of almost exclusively *T. gondii* infected cells from 12 hpi which included both strains (LDM and PTG). Interestingly, we found uniform distribution of cells 3 hpi, uninfected cells and lysate cells challenged across the remaining clusters (M1 and M3-M5) (**Figures 1D, E**; **Supplementary Figure 2**).

Further, we observed four clearly separated groups of cells, including clusters M1-M2, M3-M5, M6-M7 and M8-M9, suggesting clear transcriptional differences between them (**Figure 1D**). Single cell studies have previously enabled the investigation of bimodality in the expression of seemingly homogenous BMDC populations (9, 10). Helft and colleagues suggest that murine bone marrow cells cultivated with granulocyte-macrophage colony-stimulating factor (GM-CSF), results in a heterogeneous population of CD11c+ MHCII+ macrophages (GM-Macs) and dendritic cells (GM-DCs) (9). With Helft et al. as our reference, we were able to classify clusters M1-M2 and cluster M8 as GM-DCs and the remaining murine clusters M3-M7 and M9 as being predominantly composed of GM-Macs (**Figure 1F**, **Supplementary Figure 3**, **Supplementary Data 1**). The majority of late stage infected (12 hpi) cells can be divided into three major clusters: I) M2, containing a mixture of LDM and PTG infected GM-DCs, II) M6, containing exclusively PTG infected GM-Macs and III) M7, containing exclusively LDM infected GM-Macs (**Figure 1E, F**). This indicates that different clonal lineages of *T. gondii* elicit different host responses in GM-Macs but not in GM-DCs at 12 hpi *in vitro*.

### Different clonal lineages of *T. gondii* evoke differential host responses in a cell type dependent manner

**Figure 2:**
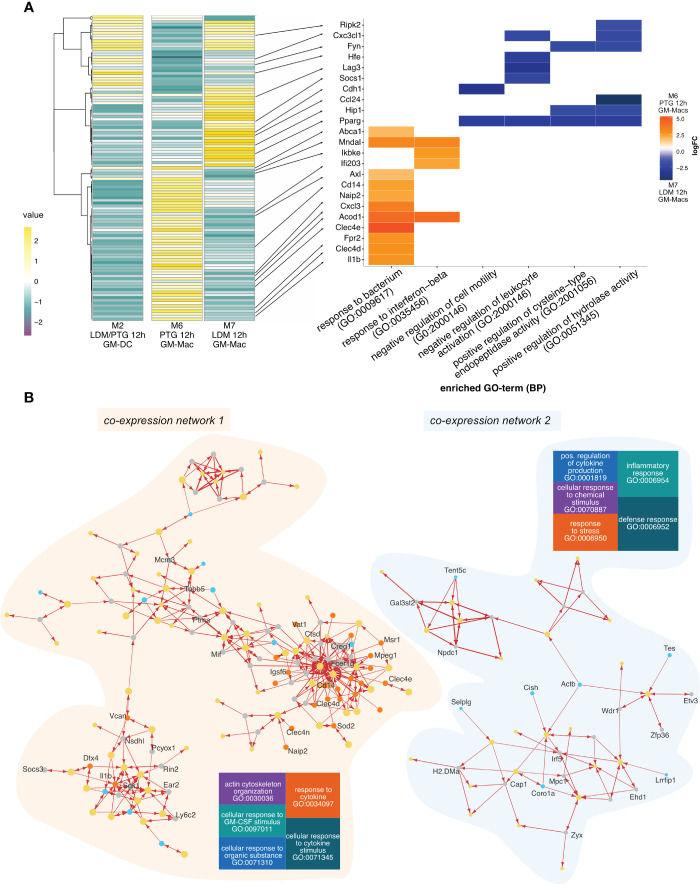
Gene expression and inter-species co-expression patterns of PTG and LDM infected BMDCs 12h post infection. **(A)** Heatmap of differentially expressed genes between cluster M6 (mainly GM-Macs containing PTG infected cells 12 hpi) cluster M7 (mainly GM-Macs containing LDM infected cells 12 hpi) across all clusters containing 12 hpi cells, including cluster M2 (mainly containing GM-DCs infected with both, LDM and PTG for 12h) (left panel). Average expression values (values) are depicted in a color scale from negative expression (purple, dark) to positive expression (yellow, light). The right panel depicts a heatplot of the logFC of selected genes (indicated with a black arrow) differentially expressed genes between the investigated groups (M6 and M7) and biological processes (GO). Positive values (orange) indicate upregulation in cluster M6 (PTG, 12 hpi). Negative values (blue) indicate upregulation in cluster M7 (LDM, 12 hpi). **(B)** Gene co-expression network of differentially expressed genes of cluster M6 (left panel) and M7 (right panel). Nodes represent genes with size representing weight. Node colors indicate gene source, with M7 marker genes in turquoise, M6 genes in orange, *T. gondii* genes in yellow and remaining *M. musculus* genes in gray. Arrows indicate the direction of co-expression. Increased arrow width indicates higher correlation value. Tree plots show the most highly enriched biological processes of the host. The size of each square represents the enrichment of each term, with larger squares exhibiting higher enrichment.

Parsing out the different transcriptional host responses of the cell subtypes across the 3 cluster groups highlighted above, we first performed a Differential Gene Expression Analysis (DGEA) between clusters M6 (PTG at 12 hpi) and M7 (LDM at 12 hpi), composed of GM-Macs. We then compared expression profiles of differentially expressed marker genes between M6 and M7 across the 3 clusters (M2, M6 and M7). Interestingly transcriptional profiles of M2 cells (GM-DCs) were more similar to cluster M7 (**Figure 2A**). Gene Ontology (GO) term enrichment revealed that genes upregulated in M6 (PTG at 12 hpi) are involved in the formation of complex signal transduction networks that activate innate immunity and inflammation (47) by the induction of immune mediators (48), Pattern Recognition Receptors (PRRs) (49), the JAK/STAT pathway (50), NF-κB signaling (51), and inflammasome activation (52). Conversely, the majority of genes upregulated in M7 (LDM at 12 hpi) are involved in the limitation of the immune and inflammatory response (53–56) and genes exhibiting balancing effects on pro- and anti-inflammatory signals (*Rip2k*, *Pparg*) (57–59).

In line with previous reports, clusters consisting of cells infected with *T. gondii* for 12h, irrespective of cell type or clonal lineage, expressed an S-phase cell cycle signature (60, 61) (**Supplementary Figure 4**). This was further validated by expression of prominent cell cycle regulators, resembling an S-Phase regulatory state in late stage infected cell clusters (**Supplementary Figure 5**). Together with the effects on expression of immune genes this observation highlights the ability of *T. gondii* to modulate gene expression of multiple independent pathways simultaneously.

Co-expression networks identify which genes tend to show coordinated expression, enabling the identification of potential regulatory genes. To investigate co-expression networks between *T. gondii* and host genes across single cells, we identified co-expressed *T. gondii* genes and genes of cluster M6 or M7 using a correlation-based approach.

The network analysis revealed several co-expression clusters of variable size (**Supplementary Figure 6**). For simplicity, we selected the two largest clusters, containing the highest numbers of M6 upregulated genes (network 1) or M7 upregulated genes (network 2) for further analysis (**Figure 2B**; **Supplementary Data 3**). We observed a higher degree of connectivity for network 1 compared to network 2, indicating differential effects of infection on the gene expression program between cells infected with PTG (M6) and LDM (M7) parasites.

#### Network 1 (M6, PTG)

Overall, host genes present in interaction network 1 are predominantly involved in cytokine response and stimulation (GO:0034097, GO:0071345) and to a lesser extent, in cellular response to organic substances, GM-CSF stimulation and actin cytoskeleton organization (GO:0071310, GO:0097011, GO:0030038) (**Figure 2B**). We also observed co-expression of several marker genes from cluster M6, including *Clec4d*, *Clec4e*, *Cd14* and *Mpeg1* with a small set of *T. gondii* genes (**Figure 2B**). Based on the involvement of these host genes on immune related processes we speculate that the expression of a specific, small set of *T. gondii* genes of network 1 may be sufficient to modulate some of these processes.

#### Network 2 (M7, LDM)

Most genes of network 2 are involved in defense and inflammatory response (GO:0006952, GO:0006954), response to stress, cellular response to chemical stimulus and positive regulation of cytokine production (GO:0006950, GO:0070997, GO:0001819). Host genes in network 2 included *Tes*, *Tent5c*, *Coro1a*, *Selp1g* as well as *Cish*, *H2-Dma* and *Lrrfip1*. These genes are involved in partially opposing functions for inflammatory and immune responses as well as proliferation and tumor suppression (62, 63).

### scDual-Seq enables the identification of putative *T. gondii* genes involved in host-pathogen interactions during late-stage acute infection

We continued to investigate parasite genes co-expressed in each network elucidating on coordinated gene expression between the host and parasite. We used an additional correlation-based approach to expand the number of co-expressed *T. gondii* genes involved in coordinating gene expression of network 1 (characterized by PTG parasite infection) and network 2 ((characterized by LDM parasite infection). In brief, we selected *T. gondii* genes showing positive or negative correlation with *T. gondii* genes found in network 1 or network 2. To reduce the potentially masking effects caused by differences in expression of replication genes of the parasite, we excluded genes of the S/M and G1 sub-transcriptome described using published bulk data (64). This analysis resulted in the generation of the expanded set of co-expressed *T. gondii* genes of network 1 (ETGN1) and the expanded set of co-expressed *T. gondii* genes of network 2 (ETGN 2).

**Figure 3:**
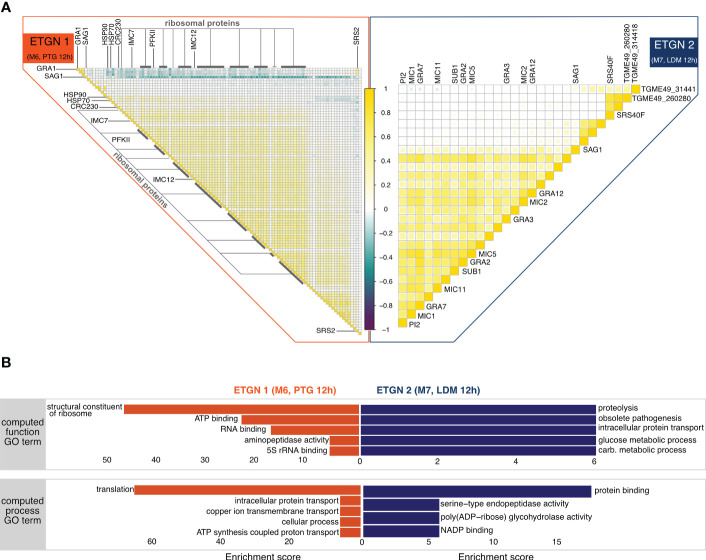
*T. gondii* differential transcription of clonal lineages during infection progression in BMDCs. **(A)** Visualization of Pearson correlation coefficients between genes in extended *T. gondii* networks (ETGNs). For *ETGN 1* (left, orange bar) and *ETGN2* (right, blue bar). Correlation values are shown in a gradient from most negative values in dark purple to most positive values in yellow (light). **(B)** Gene set enrichment of *T. gondii* genes from correlation analysis shown in a) for *ETGN 1* (left, orange) and interaction *ETGN2* (right, blue). The top panel shows enriched functions and the bottom shows enriched processes.

We observed that the majority of genes of ETGN1 exhibited positive correlation with each other and only a few anti-correlated genes. ETGN2 comprised fewer genes which showed correlation to *T. gondii* genes of network 2, when compared to the number of correlated *T. gondii* genes of network1. However, all genes of ETGN2 exhibited positive correlation with one another, indicating these genes tend to be expressed together (**Figure 3A**, **Supplementary Figures 7-8**, **Supplementary Data 4-6**).

#### ETGN 1 (M6, PTG)

The majority of genes in ETGN 1 (PTG, 12h) belongs to the group of ribosomal proteins. We observed highest correlation values between ribosomal protein genes and genes important for the inner membrane complex of *T. gondii*. We observed a negative correlation between two members of the SRS (SAG1-related sequences) superfamily, namely *SAG1* and *SRS2*, and the majority of the remaining ETGN 1 genes. SRS proteins have been linked to host cell attachment and activation of host immunity to regulate virulence (65). Our observations indicate that ribosomal protein genes and SAG-related sequences may fulfill opposite tasks in the parasites (**Figure 3A**, **Supplementary Figure 7**, **Supplementary Data 4-5**).

In agreement with the observed correlations of a high number of genes encoding ribosomal proteins, we found high enrichment of structural constituents of ribosome and RNA-/5s rRNA binding function as well as translation processes. Together with the high enrichment of energy metabolism related processes and functions, this may indicate that the PTG parasite’s increased energy expenditure is necessary for survival and the potential preparation for stage differentiation processes such as the formation of bradyzoites (**Figure 3B**).

#### ETGN 2 (M7, LDM)

ETGN 2 (LDM, 12h) is characterized by positive correlation between dense granule (*GRA12, GRA3, GRA2, GRA7*) and microneme *(MIC1, MIC11, MIC5, MIC2, SUB1)* (45, 66) associated genes. Conversely to ETGN 1, we found that *SAG1* exhibited positive correlation with the remaining genes of ETGN 2 (**Figure 3A**, **Supplementary Figure 8**, **Supplementary Data 4 + 6**).

We observed enrichment of the GO terms for metabolic processes and functions, indicating that the LDM strain, which is overrepresented in ETGN 2, upregulates the macrophage energy metabolism more efficiently when compared to the PTG strain. This may indicate that LDM parasites change the macrophage environment more effectively in the favor of their growth and replication by scavenging essential nutrients from the host which are being produced at higher rates upon infection. Despite being defined as obsolete, we found it appropriate to highlight the term “obsolete pathogenesis” given its enrichment in more virulent LDM parasites. Suggesting this term may be related to virulence or host-pathogen interactions (**Figure 3B**).

### *T. gondii* parasites show differential gene expression linked to intracellular protein localization across all identified host clusters

**Figure 4:**
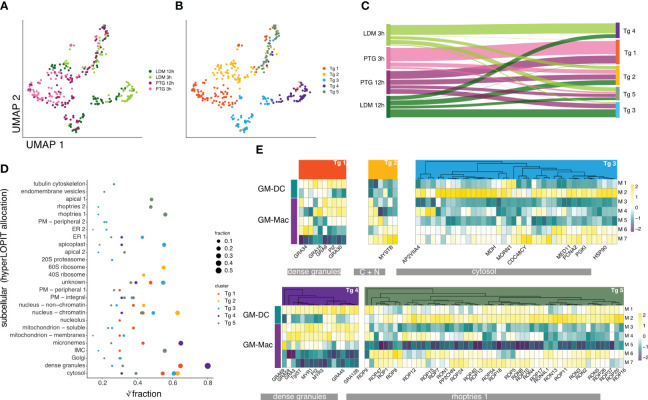
*T. gondii* gene expression profiles and differential association with subcellular localisation across parasite and host clusters. **(A)** UMAP projection of *T. gondii* infected cells grouped by identified cluster. **(B)** UMAP projection of *T. gondii* infected cells grouped by condition. **(C)** Proportion of cells of each original infection condition (left) associated with each identified cluster (right). The color of the ribbon refers to the original condition. The color of the right bar indicates the cluster association (Tg1-Tg5). **(D)** Cube root transformed Fraction of marker genes of identified *T. gondii* clusters encoding proteins located to different localizations in *T. gondii* identified previously using hyperLOPIT (67). The size of each dot refers to the non-transformed fraction of differentially expressed genes to each location on the y-axis. **(E)** Heatmap depicting differentially expressed *T. gondii* genes grouped by *T. gondii* clusters (Tg1- Tg5) across host clusters exhibiting infection (M1-M7). For each *T. gondii* cluster, genes of the highest fraction of intracellular localization were selected for comparison. *T. gondii* clusters are represented in the colors selected for each cluster. Cell type annotations of host clusters are indicated by turquoise (GM-DC) and purple (GM-Mac). Averaged gene expression is shown in a color gradient from low expression (dark purple) to high expression (yellow).

We sought to provide further detail on how the parasite modulates its gene expression in response to the host investigating exclusively *T. gondii* gene expression. In contrast to the infected host cells, *T. gondii* parasites were actively replicating.Therefore, we assigned cell cycle stages (S/M or G1) across all cells, using previously described sub-transcriptomes (64) and regressed the cell cycle out of our dataset, resulting in 5 clusters (Tg1-5) across infection conditions (**Figure 4A, B**; **Supplementary Figure 9**).

When comparing the infection conditions with the identified clusters, we show that cluster Tg 1 is characterized by PTG specific expression. Cluster Tg 2, similar to Tg 5, is characterized by the presence of parasites of both strains and timepoints. Tg 3 mainly consisted of parasites from 12 hpi, while Tg 4 consisted mainly of LDM parasites (**Figure 4C**).

To further define what is driving the differences between parasites within each cluster, we investigated whether they express organelle-specific genes by comparing our gene expression results to spatially resolved proteome data on the subcellular level (67) (**Figure 4D**). While transcription data presented here does not equal protein expression and subsequent effector activity, comparison with spatially resolved proteomics data may give an indication about what processes the parasites are preparing for, providing a glimpse into the future of the parasite’s development.

Clusters Tg1 and Tg4 showed the highest fraction of genes belonging to dense granules. Across clusters containing mixed parasite populations, Tg2 exhibits the highest fraction of genes located to the nucleus, the cytosol and the 60S ribosome as well as unknown localization. Tg3 shows similar distributions of genes across localizations as Tg 1, with higher fractions within the apicoplast and the ER (**Figure 4D**). Tg 5 generally shows the highest number of upregulated genes across all *T. gondii* clusters, (**Supplementary Figure 10**; **Supplementary Data 7**) with the majority associated with rhoptries (**Figure 4D**; **Supplementary Data 8**). Rhoptry proteins, located in the apical region of the parasite, fulfill multiple roles for virulence, host cell invasion and potentially parasitophorous vacuole formation as well as manipulation of host response (68–70). The observed differences indicate that parasites within each cluster undergo or prepare themselves for different processes during infection, for example invasion, egress, stage differentiation or immune evasion and virulence.

We further examined the relationship between parasite clusters (Tg 1-5) and their gene expression profiles and *T. gondii* infected host cell clusters (M1-7). We observed obvious differences in *T. gondii* expression profiles for markers across host clusters (**Figure 4E**; **Supplementary Figures 11, 12**). For instance, different sets of dense granule genes between Tg 1 (PTG) and Tg 4 (LDM) showed inverse expression in clusters M6 (PTG 12 hpi) and M7 (LDM 12 hpi) (**Figure 4E**). In addition to observed differences between known subtypes of host cell types (GM-DCs and GM-Macs), we observed downregulation of all rhoptry and other location markers in host cell cluster M5 (**Figure 4E**).

### Infection with *T. gondii* shares host response similarities to stimulation by CpG-containing oligodeoxynucleotides (cpGB) and other immune stimulating molecules

Our findings suggest that *T. gondii* LDM (LDM) and PTG (PTG) provoke differential host cell responses primarily in GM-Macs and that an extended network of *T. gondii* specific genes are involved in these host-pathogen interactions at the single cell level. Beyond, we wanted to explore which innate immune responses are involved in the differential reactions of the two clonal lineages, comparing host responses to other common microorganisms.

**Figure 5:**
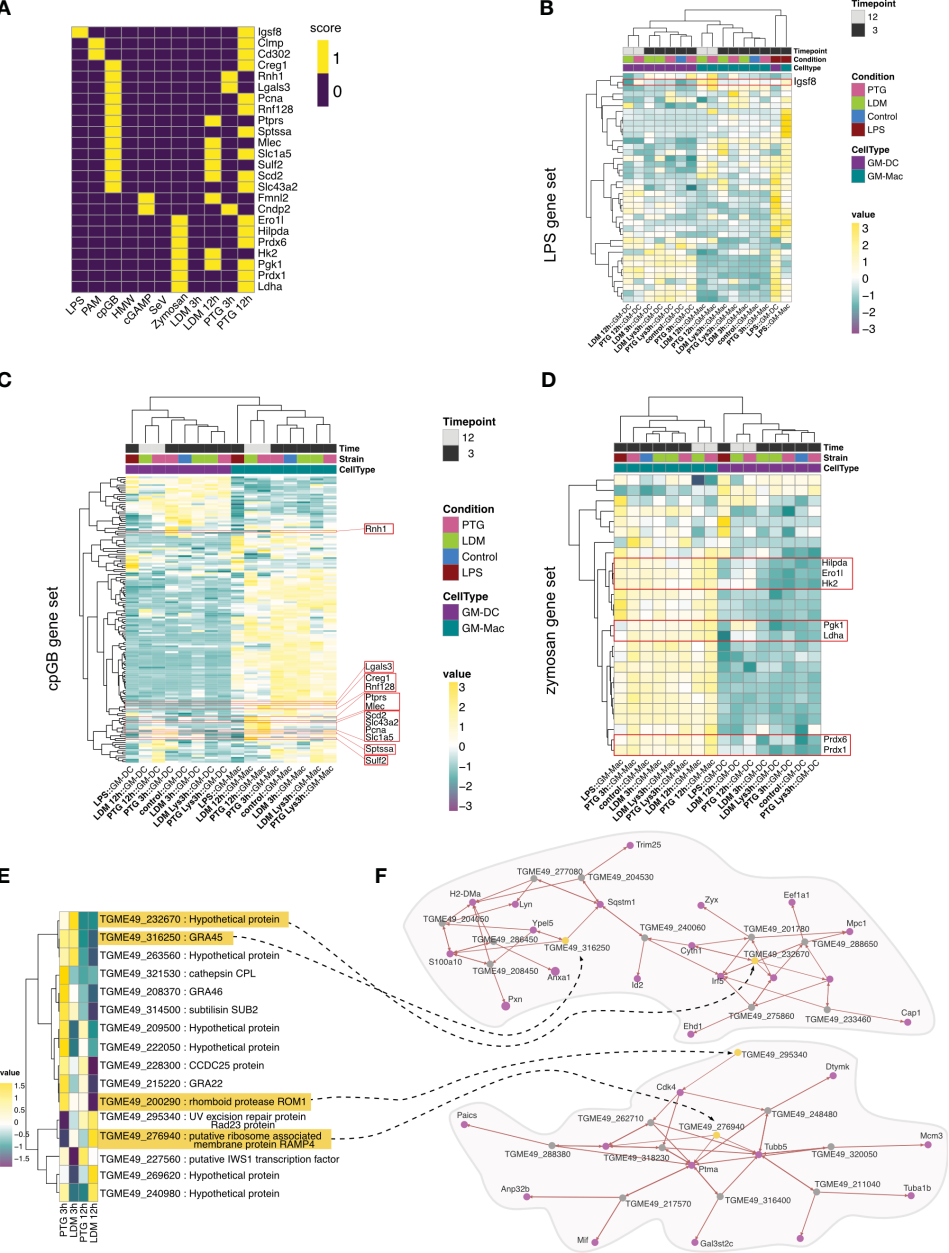
Cell type differentiation and comparative analysis of response to *T. gondii* infection with established PAMPs and *T. gondii* INFγ challenge. **(A)** Heatmap depicting commonly expressed genes between BMDCs exposed to established PAMPs (LPS, PAM, cpGB, HMW, cGAMP,SeV and Zymosan) and cells infected with *T. gondii* LDM and PTG for 3 or 12 hours. A score of 1 (yellow) indicates differential expression of the gene in the respective condition while a score of 0 indicates no differential expression. **(B)** Heatmap depicting expression of genes upregulated in LPS treated BMDCs (71), hierarchically clustered by condition in both cell types and gene expression. Expression values are shown in a gradient stretching from negative expression (dark purple) to positive expression (yellow). The red box highlights expressions of *Igsf8*. **(C)** Heatmap exhibiting gene expression values for previously identified cpGB signature and **(D)** zymosan signature. Shared marker genes between the reference signature (71) and our data are highlighted in red boxes. **(E)** Heatmap visualizing conducted comparative analysis between genes identified to be essential during INFγ stimulation in BMDMs (72) and their expression profiles in *T. gondii* infective states in our study (LDM and PTG infection 3 and 12 hpi). **(F)** Co-expression networks for essential *T. gondii* genes. Genes exhibiting correlation are connected with a black dashed arrow and highlighted in yellow. Correlating host genes are depicted as purple nodes. Remaining *T. gondii* genes are shown as gray nodes. The arrow indicates the direction of the correlation. Increased arrow width indicates higher correlation value.

We utilized RNA-seq data from a recent publication by Pandey and colleagues in which they studied the immune response towards seven common microbial signals in BMDCs (71). We observed the highest overlap in upregulated genes stimulated with CpG DNA type B (cpGB) and depleted zymosan, a β-glycan found in fungal cell walls, in macrophages and dendritic cells in both clonal lineages (**Figure 5A**). cpGB and zymosan are known to target TLR9 (73) or TLR2 and Dectin-1 (74). Further, we found cells infected with PTG parasites to exhibit higher overlap of expressed marker genes across most other microbial stimuli (**Figure 5A**). We found *Igsf8* as the only shared marker gene between LPS stimulation and PTG parasite infection. Upon closer inspection, we find *Igsf8* expression in both: PTG parasites and to a lesser extent in LDM infected cells. Overall we observed clear differences in expression based on cell type (GM-DC or GM-Mac), and higher expression for some genes in GM-Macs infected with PTG parasites (**Figure 5B**).

For cpGB- and zymosan-stimulated BMDCs, we observed higher expression of genes in GM-Macs in comparison to GM-DCs, irrespective of infection. Of those genes that were shared between cpGB challenge and *T. gondii* infection, upregulation was more prominent in PTG infected cells (**Figure 5C**).

We then sought to use a similar approach to compare *T. gondii* expression in our cell population to other relevant cell population data. Therefore, we employed publicly available data from *in vitro* CRISPR-Cas9 screens of *T. gondii* genes comparing it to our dual-scSeq data. When we intersected *T. gondii* marker genes of our infected conditions with high confidence hits for “fitness under INFγ stress” identified by Wang et al. (72), we found the majority of intersecting genes to be upregulated in parasites 3 hpi, with PTG strains upregulating a higher number of genes compared to LDM strains. We also found genes TGME49_276940 and TGME49_269620, exclusively upregulated in LDM parasites 3 hpi or 12 hpi, respectively (**Figure 5D**).

To further characterize interactions between *T. gondii* INFγ stress marker genes and host genes, we generated co-expression networks, resulting in multiple networks of variable sizes (**Supplementary Figure 13**). For the two largest networks, we found *GRA45* (*TGME49_316250*), upregulated in PTG parasites at 3 hpi, to be co-expressed with genes reported to be involved in regulation of innate immune response and inflammation (75, 76) or autophagy (77) (**Figures 5E, F**). Conversely, network genes upregulated in LDM parasites showed co-expression with genes involved in filamentation and pathogenesis (78, 79), respectively (**Figure 5F**).

## Discussion

In this study, we performed scDual-Seq of BMDCs infected with two phenotypically distinct clonal lineages of *Toxoplasma gondii*. We analyzed infection dynamics over two timepoints (3 and 12 hpi) during acute *T. gondii* infection and described expression patterns of individual populations of host cells in tandem with *T. gondii* gene expression. Information on heterogeneity of host-pathogen interactions was lost in previous bulk-RNA sequencing studies on *T. gondii* infected BMDCs. By using a scSeq approach we were able to show that the two investigated parasite lineages manipulate two subpopulations of infected BMDCs, namely, GM-Macs and GM-DCs differently (30–32).

Our data indicates that only the GM-Mac subpopulation at 12 hpi showed distinct differential expression profiles based on the infecting parasite strain, suggesting that they are either more susceptible to strain specific modulation by PTG and LDM parasites or that the GM-Mac subpopulation responds uniquely to different parasite strains. This highlights the importance of considering transcriptional heterogeneity of host-pathogen interactions, previously suggested by single cell studies on human monocytes infected with *T. gondii* parasites (36). Patir et al, investigate single-cell infection dynamics of human monocytes at 1 hpi, elaborating in detail on transcriptional heterogeneity and chemokine secretion. Our study significantly augments existing data on this important topic, by investigating transcriptional differences between different subpopulations of host cells infected with parasite strains of different genetic background and including the corresponding parasite expression dynamics.

Upregulation of genes associated with anti-inflammatory effects in LDM parasite infected GM-Macs as opposed to upregulation of a pro-inflammatory response in PTG infected cells is in agreement with previous studies in murine macrophage infections (30). The higher similarity of the gene expression signature of anti-inflammatory signals in GM-DCs infected with *T. gondii* parasites of the LDM as well as the PTG strain, suggests that the parasite exploits or even promotes immune tolerogenic capacities of infected DCs. DCs represent important regulators of the balance between tolerance and immunity, giving rise to large numbers of subtypes partially exhibiting immune tolerogenic phenotypes (80, 81). Moreover, stimulation with GM-CSF in BMDCs was shown to favor immune tolerogenic DC populations (82, 83). This suggests that, in addition to the reported manipulation of the migratory behavior of DCs promoting parasite dissemination (20), the parasite may be more efficient in generating a suitable environment for replication and survival in GM-DCs in comparison to GM-Macs. Conclusively, our observations highlight the importance of considering heterogeneity in host-pathogen interactions and prompt more detailed studies of the interactions between *T. gondii* and BMDCs, particularly GM-DCs.

We generated and examined co-expression networks of host- and parasite gene expression based on differential marker genes of cluster M6 (PTG, 12h) and M7 (LDM, 12h). We interpret the higher connectivity in *network 1* to be indicative of an increased response to parasite expression in PTG parasites at 12 hpi in infected GM-Macs. Genes exhibiting strong correlation with only a few parasite genes within the network included C-type lectins *Clec4d*, *Clec4e* and *Clec4n*, important players in innate recognition of pathogens and interleukin production as well as phagocytosis of parasites in macrophages (48, 84, 85). Co-expression of these genes in network 1 may be crucial cues for the formation of bradyzoites in PTG parasites (86) as opposed to LDM parasites, which are less likely to form mature bradyzoites (87).

Genes within network 2 involved in immune system processes included genes with implications in host cell modulation of macrophages (88) or the restriction of effector T cell responses in acute viral infection (89). This supports the hypothesis that *T. gondii* LDM parasites modulate host cell responses and potentially limit them more effectively than PTG parasites. Apart from immune system related genes we also find a number of genes involved in tumor suppression including *Tes* (62) and *Tent5c* (63). This indicates that *T. gondii* LDM infection might have a more global effect on host cell proliferation and cell cycle dysregulation than reported so far.

The observed positive correlation between many genes encoding ribosomal proteins and a large number of host genes involved in energy expenditure, phagocytosis and inflammation in extended *T. gondii* network 1 (*ETGN 1*) suggests that the majority of parasites may express these genes in response to the pro-inflammatory immune response of the infected host cells. Moreover, the highly expressed ribosomal proteins may represent an interesting pool of potent targets for the innate immune system. For instance, ribosomal protein P2 has been previously identified as a target for protection against toxoplasmosis (90). Based on previous reports of the importance of MIC1, MIC2 and GRA2 for in effective immune evasion or virulence (45, 91, 92), *T. gondii* genes encoding secreted proteins in *ETGN 2* likely belong to a network of genes with importance for these processes. Conclusively, while not providing causality or evidence for direct interactions, which is beyond the scope of this study, we consider our findings a valuable resource to investigate potential direct interactions between genes that correlate positively in expression in single cells for future functional studies on host-pathogen interactions.

Early stage parasites of both lineages showed expression of dense granules and micronemes, known to be important during cell adhesion and invasion of the parasite (93). Recent studies suggest that dense granules affect the behavior of host cells directly, as shown for altered cell-motility by GRA28 in infected macrophages (21).

The overall high fraction of dense granule genes at 3 hpi and the differential expression of these genes between LDM and PTG parasites further support the importance of dense granules for host-pathogen interactions and potential strain-specific host-cell modulation during the subsequent parasite development. Comparing the expression of location specific genes across host-cell clusters, we observed the absence of all genes encoding for rhoptry proteins in one sub-cluster of GM-Macs. Multiple rhoptries have been implicated in virulence and modification of host response, such as ROP16 and ROP18 (70, 94), indicating that parasites in this sub-cluster are less effective in establishing virulence, potentially being successfully targeted by the immune system. It is important to restate that transcription data presented here does not equal protein expression and subsequent effector activity. Therefore, our data draws an image of the potential future development of the parasite within a host-cell.

Comparing gene expression of *T. gondii* infected host cells to expression profiles of cells stimulated with a variety of PAMPs, we found the highest expression similarities to cpGB as well as zymosan stimulation, primarily in PTG infected GM-Macs 12 hpi. Zymosan stimulation results the secretion of pro-inflammatory cytokines (TNF-alpha, IL-8) and activation of the Dectin-1/Syk/NF-B signaling pathway, mediating an inflammatory response *via* TLR2 (95). CpG motifs lead to the activation of the NF-B-REl dependent innate pathway (96) *via* TLR9. *T. gondii* infection stimulates INF production and pro-inflammatory cytokine production in a Myeloid Differentiation factor 88 (MyD88) dependent manner. It is proposed that this pathway is mainly activated through stimulation of TLR11 (44, 97). However, there is additional evidence of the involvement of TLR9 (97, 98) and TLR2 (97, 99), confounded by similarities in expression patterns in our data. Moreover, our data provides gene lists of important genes expressed during a potential TLR2 or TLR9 stimulation by *T. gondii* parasites of different clonal lineages. This further supports the notion of the onset of a stronger more pro-inflammatory immune response by *T. gondii type* II (PTG) parasites in comparison to type I (LDM) parasites.

Co-expression analysis revealed genes involved in modulation of innate immune responses and TLR function are co-expressed with dense granule gene, GRA45, implying a potential role in immune modulation during an inflammatory response of the host. In addition, co-expression of genes related to cytoskeleton formation and pathogenesis as well as cell cycle regulation, supports the diversity of host cell modulation by *T. gondii*.

In summary, this study provides a detailed resource of host-pathogen interactions over the time-course of an acute *T. gondii* infection in BMDCs. The identification of differential responses in distinct sub-populations of cells highlights the importance of studying infection kinetics at the resolution of single infected cells and raises the question how and why parasites of different clonal origin modulate cell types differently or change cell type signatures. We show co-expression of parasite ribosomal proteins and pro-inflammatory genes in PTG infected cells and of dense granules and immune modulating genes in LDM infected cells, highlighting unprecedented differential modulation of host-pathogen interaction pathways between clonal lineages at the single-cell level. Further, we show differential expression of rhoptry, dense granule and microneme genes between PTG and LDM parasites as well as clusters of host cells, with strain specific host modulation by dense granules and micronemes contrasting rhoptry expression patterns. This highlights the importance of dense granules for nuanced host-pathogen interactions, prompting future functional studies on these effectors. Moreover, the integration and comparative analyses between our data and previously published data highlights the possibility of validating and identifying new expression programs during host-parasite interactions.

We anticipate that our observations provide information of future clinical implications, such as the development of effector molecule-targeting therapies, by providing novel candidate genes identified both in the host and parasite. We further anticipate that the data presented here provides a unique and comprehensive resource for studies of host-parasite interactions between *T. gondii* parasites and infected immune cells. Finally, this resource will serve as a tool, which can be applied to study host-pathogen interactions on a variety of other invasive pathogen and host cell complexes of medical and veterinary importance, including *Plasmodium*, *Cryptosporidium*, *Babesia*, and *Leishmania* as well as different bacteria and viruses.

## Methods

### Cell culture

Primary bone marrow-derived mononuclear phagocytes were generated as previously described (100, 101). Briefly, cells from the bone marrow of 6-10 week old C57BL/6 mice (Charles River) were cultured in RPMI 1640 (Gibco) complemented with 10% fetal bovine serum (FBS, Sigma), gentamicin (20 μg/ml; Gibco), glutamine (2 mM; Gibco) and HEPES (0.01 M; Gibco), referred to as complete media, and further supplemented with 10 ng/ml of recombinant mouse granulocyte-macrophage colony stimulating factor (GM-CSF) (Peprotech). Culture media was replenished on days two, four and six. Loosely adherent cells (102) were harvested on day seven or eight for experiments.

The parasite lines used include GFP-expressing RH-LDMluc (LDM, cloned from RH-GFPS65T) and GFP-expressing PTGluc (PTG, cloned from ME49/PTG-GFPS65T) (103). Tachyzoites were maintained by serial 2-day passaging in human foreskin fibroblast (HFF-1 SCRC-1041, American Type Culture Collection) monolayers cultured in Dulbecco’s modified Eagle’s medium (DMEM; Gibco) with 10% FBS, gentamicin (20μg/ml), glutamine (2mM), and HEPES (0.01 M).

### Infection challenges, lysates and LPS

On day seven or eight, BMDCs in complete media were challenged with freshly egressed *T. gondii* tachyzoites. To ensure an infection-rate of single parasites above 80%, cells at 3 hours were infected at a multiplicity of infection (MOI) of 3 (3h), or MOI of 1.5 (12 h) before single cell sorting. Under these conditions, infection frequencies were > 50%, with negligible cell lysis and >80-90% of infected cells containing a single *T. gondii* vacuole. Negative controls were treated with parasite lysates or not stimulated, while positive controls were treated with 100ng/ml LPS for 3 hours. The parasite lysates were produced by ultrasonication of live tachyzoites, where ultrasonication was applied 3 times at 10 second intervals with 1-minute interval on ice.

Post-infection, cells were treated with Fc-block (anti-mouse CD16/CD32 antibody, 1:200; BD 553142) for 15 minutes, washed with D-PBS and further incubated with a monoclonal antibody against CD11c conjugated with PE-Cyanine7 (1:200; Thermofisher 25-0114-82) for 30 minutes, washed with D-PBS and resuspended in ice-cold D-PBS. Cells were kept on ice prior to sorting.

### Cell sorting and library preparation

A preliminary trial was set up to validate optimal cycling conditions for cDNA amplification of single *T. gondii-*infected BMDCs by performing rt-qPCR of target genes known to be expressed in *T. gondii* and genes known to be expressed in DCs (primer sequences).

The sorting was performed with a MoFlo Astrios EQ (Beckman Coulter, USA) cell sorter using 488 and 532 nm lasers for excitation, 100 µm nozzle, sheath pressure of 25 psi and 0.1 µm sterile-filtered 1 x PBS as sheath fluid. Flow sorting data was interpreted and displayed using the associated software, Summit v 6.3.1.

To test the precision of the adjustments made to center the drop in each well, a colorimetric test mimicking the sort was done based on (104). A 1.5 mg/µl solution of HRP (cat no 31490, ThermoFisher Scientific) with 1 drop of flow check beads (Beckman Coulter, USA) was sorted into each well of an Eppendorf 384-well plate (Cat no 34028, ThermoFisher Scientific). A color change after sorting indicated that the drop hit the sort buffer and that the precision was adequate.

Individual BMDCs or single *T. gondii* infected BMDCs were deposited into 384-well plates (Eppendorf twin.tecTM PCR plates) containing 2.3 µl of lysis buffer (105) using a CyClone™ robotic arm and at highly stringent single cell sort settings (single mode, 0.5 drop envelope). Side scatter was used as the trigger channel and sort regions were based on *Toxoplasma* cells expressing GFP and the surface antibody CD11C-PE-Cy7 bound to human dendritic cells. GFP was excited at 488 nm and detected with a 530/40 nm bandpass filter whereas PE-Cy7 was excited at 532 nm and detected using a 695/70 nm bandpass filter. The plate and sample holder were kept at 4 °C at all times during the sort. After the sort, the plates were immediately spun down and put on dry ice.

Single BMDCs or single *T. gondii* infected BMDCs were sorted directly into lysis buffer and cDNA libraries were generated using a slightly modified version of Smart-seq2 as previously described (105), but where we used 20 cycles for cDNA amplification.

### Single cell sequencing

Single-cell libraries were sequenced at the National Genomics Infrastructure, SciLifeLab Stockholm, using the HiSeq2500 platform (Illumina) for 56 bps single-end sequencing. We sequenced a total of 764 (negative controls n=2, per plate) single BMDCs or *T. gondii* infected BMDCs from a total of 8 different conditions.

### Computational analysis

#### Mapping and annotation and filtering

A custom reference genome was made by combining *Mus musculus* GRCm38 and *Toxoplasma gondii* TGA4.44. The reads were aligned to the genome using STAR v 2.7.2, and gene expression was measured using featureCounts v 2.0.0, using default settings. Cells with less than 10 000 mapped reads were filtered due to substantially inferior quality and the remaining 518 cells were subjected to subsequent computational analysis. A total of 62 276 genes across 532 cells for the host (*Mus musculus)* and *T. gondii* were analyzed.

#### Normalization, dimensionality reduction and clustering

Main computational analysis of read-count matrices was performed using the Seurat package (v 4.0.3) (106) in R (v 4.2.0). The complete R workflow can be assessed in an R markdown (see code availability section). First, count matrices and metadata were loaded and split by the respective species. Ensembl IDs of genes were translated to gene symbols and cells with a mitochondrial gene count above 10% were filtered. Subsequently reads were normalized for sequencing depth using the “SCTransform” function in Seurat, selecting the top 3000 variable genes (107). Thereafter, dimensionality reduction was performed using PCA, computing the first 50 PCs. The first 15 PCs from the analysis were then subjected to shared-nearest-neighbor (SNN) inspired graph-based clustering *via* the “FindNeighbors” and “FindClusters” functions. For modularity optimization, the louvain algorithm was used and clustering was performed at a resolution of 0.8 for clustering granularity, resulting in 9 clusters. After clustering, a UMAP dimensionality reduction was performed.

#### Differential gene expression analysis

Differential gene expression analysis (DGEA) of genes in identified clusters was performed using the function “FindAllMarkers” from the Seurat package (v. 4.0.5). Following the default option of the method, differentially expressed genes for each cluster were identified using a non-parametric Wilcoxon rank sum test. Differentially expressed genes in a cluster were defined by setting initial thresholds above a logarithmic fold-change of 0.5 and being present in at least 25% of the cells belonging to the same cluster. Representative marker genes with an adjusted *p*-value below 0.05 for each cluster were further selected. *p*-values were adjusted using a Bonferroni correction including all genes in the dataset. To find representative marker genes with elevated expression in comparison to the remaining clusters, only positive log fold-changes were considered. For individual analyses such as gene enrichment analysis (see “Gene set enrichment analysis (gsea)”), threshold values for differential gene expressions were modified and will be described in detail in the respective sections of the materials and methods and results. To identify DEGs between specific clusters of interest, the “FindMarkers” function in Seurat was used and the identities were set to the respective clusters of interest. The same thresholds as stated above were used to define DEGs.

For visualization purposes of DGE data, the gene expression data for all cells was averaged and grouped according to their cluster identity, resulting in average expression of each gene and cluster. Then, expression data was scaled and clipped to average expression values between -1.5 and 1.5, with negative values representing downregulation and positive values representing upregulation of each gene.

### Cell type classification and annotation

We classified cell types in our data using the clustifyr package (v.1.8.0) (108) and the sorted microarray expression data presented in (9) as reference. In brief, clustifyr adopts correlation-based methods to find reference transcriptomes with the highest similarity to query cluster expression profiles. After converting the microarray data in the correct format for automated cell type annotation, we used the default settings (Spearman rank correlation) to estimate correlation coefficients between the single cells in our clusters and the reference cell types.

### Cell cycle scoring

To chart the cell cycle phases of individual cells, cell cycle scoring was performed using the “CellCycleScoring” function in Seurat. Scoring was performed based on the host (*M. musculus*) expression data as well as on the *T. gondii* data. Here the scoring function was performed using the default parameters. In brief, the function assigns each cell a score based on its expression of G2/M and S phase markers, which are assumed to be anticorrelated in their expression levels. Thus, cells expressing neither are assumed to be in G1 phase. Cell cycle associated marker gene lists for mouse were retrieved from: https://raw.githubusercontent.com/hbc/tinyatlas/master/cell_cycle/Mus_musculus.csv (20210707). These gene lists are based on orthology analyses of human cell cycle genes presented in (109).

*T. gondii* cell cycle associated genes were extracted from supplementary data of (64). As the cell cycle stages of *T. gondii* contain a S/M and G1 phase only, the resulting phases were changed accordingly.

### Gene set enrichment analysis (GSEA)

To interpret gene expression data and differentially expressed genes further, we performed a gene set enrichment analysis for clusters of interest. This was done using the gseGO function of the clusterProfiler package in R (v.4.0.5). To identify significantly enriched genes, we set the ontology parameter to use gene set enrichment for biological processes and set the gene set parameters to include a minimum of three genes and a maximum of 800 genes in one set. The reference dataset for *M.musculus* was used, and the resulting p-value from the fast preranked gene set enrichment analysis (fgsea) was adjusted using a Benjamini-Hochberg correction. For differentially expressed genes between cluster M6 and cluster M7, correction of gene set enrichment resulted in non-significant p-values, which is why we don’t report significant differences for gene set enrichment but only fold changes of genes of interest with their respective biological process (**Figure 2A**).

To identify *T. gondii* specific gene set enrichment we used the ToxoDB database to annotate all *T. gondii* genes present in our dataset and included computed GO terms for I) function and II) process generated by VeuPathDB utilizing InterPro-to-GO (110). Then the counts for each computed GO-term within the gene list of interest were determined. After the fraction of genes of the geneset of interest was determined and divided by the fraction of the reference (all data) to determine the enrichment for each term and geneset. We termed the resulting value “enrichment score” in our data.

### Comparative analysis of gene expression and reference data

For reference data integration and comparison we included three publicly available datasets, to I) perform cell type annotations (cell type classification and annotation) (9) II) to compare the response in our data to data investigating PRR agonists (71) and III) to compare our results to identified essential genes for *T. gondii* during INFγ stress (72). For II) we first identified differentially expressed genes for each single condition (PAMP) using DESeq2 v.1.36.0 (111). We investigated expression patterns for II) by intersecting genes identified to be upregulated in BMDCs during stimulation with each agonist. For simplicity we generated bimodal scores resulting in two modes: expression (1) and no expression (0). We further show gene expression of PRR agonists by investigating aggregated expression of individual genes across our experimental conditions, visualized in a heatmap. We performed similar analyses for III), additionally investigating co-expression of shared genes between the reference data and our gene expression data.

### Co-expression network analysis

To investigate inter-species co-expression networks we first performed an asymmetric Biweight midcorrelation (bicor) to identify gene expression correlations between *T. gondii* and *M. musculus* genes across cells. Using a k-nearest neighbor approach, we defined the top 20 interactors for each gene in the *T. gondii* expression matrix and *M. musculus* expression matrix. Using igraph (v.1.3.1), we then constructed correlation network plots (Co-expression networks). Using the three nearest neighbors (interactors), a correlation threshold of 0.3/-0.3 and 3 steps. The number of steps defines how many additional interactors after the primary interaction should be displayed after the interactors of the provided seed genes. We displayed the largest interaction clusters using the “induced_subgraph” function in igraph. To integrate publicly available data on *T. gondii*, we investigated co-expression of *T.gondii* genes, which were previously shown to be essential during INFγ stress in BMDMs (72).

### Generation of extended *T. gondii* co-expression

To investigate gene expression correlation between genes resulting from the co-expression network analysis and other genes in the dataset, we first performed an asymmetric bicor correlation analysis between all genes present in the co-expression network cluster of interest and all remaining genes to determine which genes exhibit positive or negative expression correlation across cells. Then, we decided for an appropriate cut-off, only considering genes to be positively or negatively correlated based on the distribution of correlation values, resulting in thresholds between 0.15/-0.15 and 0.25/-0.25. After identifying a subset of genes exhibiting correlation above or below the determined threshold, we calculated pearson correlations between genes of interest and the subset of correlated genes and visualized them using the corrplot package (v.0.92).

## Data availability statement

The datasets presented in this study can be found in online repositories. The names of the repository/repositories and accession number(s) can be found below: PRJNA918538 (SRA). The code used for the analysis is available on Github via https://github.com/ANKARKLEVLAB/t.gondii_bmdc upon publication.

## Ethics statement

The Regional Animal Research Ethical Board, Stockholm, Sweden, approved experimental procedures and protocols involving extraction of cells from mice (N135/15, N78/16), following proceedings described in EU legislation (Council Directive 2010/63/EU).

## Author contributions

JA, JH and ABa conceived the study. JA and JH supervised the study. ABh cultured cells and performed infections. JA and A-MD performed sorting and scSeq. MM and JH performed mapping and initial computational analyses. FH performed computational analysis of the data and generated figures. FB and FH designed and implemented co-expression network analysis; MM generated the shinyapp FH, AD and JA wrote the manuscript. All authors read and reviewed the manuscript.
